# The relationship between women’s body mass index and breast cancer outcomes was U-shaped

**DOI:** 10.3389/fonc.2023.1191093

**Published:** 2023-05-25

**Authors:** Wensong Wei, Suosu Wei, Zhen Huang, Qiuhuan Zhang, Fei Liu, Yujie Xie, Jing Wei, Chongde Mo, Ying Zhou, Shengpeng Qin, Quanqing Zou, Jianrong Yang

**Affiliations:** ^1^ Department of Breast and Thyroid Surgery, Guangxi Academy of Medical Sciences, People’s Hospital of Guangxi Zhuang Autonomous Region, Nanning, Guangxi, China; ^2^ Department of Scientific Cooperation of Guangxi Academy of Medical Sciences, People’s Hospital of Guangxi Zhuang Autonomous Region, Nanning, Guangxi, China; ^3^ Department of Colorectal and Anal Surgery, Guangxi Academy of Medical Sciences, People’s Hospital of Guangxi Zhuang Autonomous Region, Nanning, Guangxi, China; ^4^ Research Center of Medical Sciences, The People’s Hospital of Guangxi Zhuang Autonomous Region, Guang-xi Academy of Medical Sciences, Nanning, Guangxi, China

**Keywords:** breast cancer, body mass index, prognostic factor, survival, u-shaped association

## Abstract

**Background:**

Several studies have analyzed the relationship between body mass index (BMI) and the prognosis of breast cancer (BC). However, whether their relationship is linear or curvilinear remains unclear. This cohort study examined the specific relationship between BMI and BC outcomes.

**Methods:**

This retrospective cohort study included 1049 BC patients from March 7, 2013 through December 31, 2019 in a hospital. Kaplan-Meier curves, multivariate Cox proportional models, and restricted cubic spline (RCS) was used to analysis the relationship between BMI and overall survival (OS) and breast cancer-specific survival (BCSS) was analyzed.

**Results:**

During a median of 4.87 (IQR:3.26-6.84) years of follow-up period, 71 patients (6.77%) died, of which 50 (70.42%) were attributed to BC. RCS analysis revealed a U- shaped relationship between BMI levels and OS and BCSS after adjusting for other variables. The turning points of the U-shaped curves were 23 kg/m2. On the left side of the turning point, the risk of OS (HR, 0.83; 95% CI, 0.70, 0.98) and BCSS (HR, 0.80; 95% CI, 0.65, 0.98) were adversely correlated with BMI. In contrast, to the right of the turning point, the risk of OS (HR, 1.22; 95% CI, 1.10, 1.37) and BCSS (HR, 1.28; 95% CI, 1.13, 1.46) was positively related to BMI. Kaplan-Meier curves and multivariate Cox regression analyses shown consistent results with RCS analyses.

**Conclusion:**

BMI was an independent prognostic factor for BC, and had a U-shaped relationship with OS and BCSS. Interventions should be designed to improve patient outcomes based on BMI.

## 1 Introduction

According to the National Cancer Institute, about 2.3 million new instances of breast cancer (11,7%) will be reported globally in 2020, overtaking lung malignancies (11,4%) as the most often diagnosed cancer, and its prevalence is rising (1). For many years, researchers have been very interested in the assessment of BMI, trying to determine how it affects patient prognosis. Back in 2000, researchers demonstrated that BMI was an independently significant prognostic factor for postmenopausal BC and that the risk of breast cancer could be reduced through weight control and a healthy lifestyle (2). In subsequent studies, it was found that higher BMI led to worse breast cancer outcomes and that younger and node-positive patients were at greater risk (3). It is possible that part of this difference is due to obesity leading to a late diagnosis, but BMI also has a substantial influence on patients’ survival (3). In general, small tumors in large breasts may be more difficult to detect. In addition, overweight and obese women have poor compliance with healthy habits (3–5). The stigma of obesity will lead to fear, fatalism, alienation, inferiority, and embarrassment, all of which will lead to reduced compliance with screening and treatment guidelines (4, 5).Furthermore, among Asians and Africans, high BMI has been associated not only with postmenopausal breast cancer incidence, but also with premenopausal cancers (6). It is now becoming increasingly apparent that BMI can also influence the prognosis and long-term survival of cancer patients (7). Although it has been established that BMI is an essential outcome factor in breast cancer (8, 9), some studies remain controversial about this finding. In a study and analysis of data from the breast cancer population, Tan Xin et al. concluded that the prognosis of breast cancer was not influenced by BMI (10). A retrospective analysis of 418 cases of triple-negative breast cancer (TNBC) revealed that OS or recurrence-free survival (RFS) was not related to BMI (11). Another retrospective analysis of 501 TNBC patients attending the University of Washington Breast Oncology Clinic in the USA showed that neither diabetes nor BMI had an effect on survival outcomes in women with TNBC treated at an academic medical center (12). Related studies have shown that BMI affects the prognosis of breast cancers that are estrogen-dependent, while no association can be hypothesized with a rational background in estrogen-negative breast cancer (13, 14). The mechanisms underlying the link between BMI and BC prognosis are well studied and well known, but the relationship between BMI and BC prognostic curves has been rarely studied. In this study, we retrospectively analyzed data from 1049 patients with complete data retained from a single institution. The aim was to study and explore the risk factors affecting BC prognosis and to analyze the correlation between BMI and BCSS and OS to provide a reliable basis for good prognosis in the clinical management of BC patients.

## 2 Materials and methods

### 2.1 Study design

**Figure 1:**
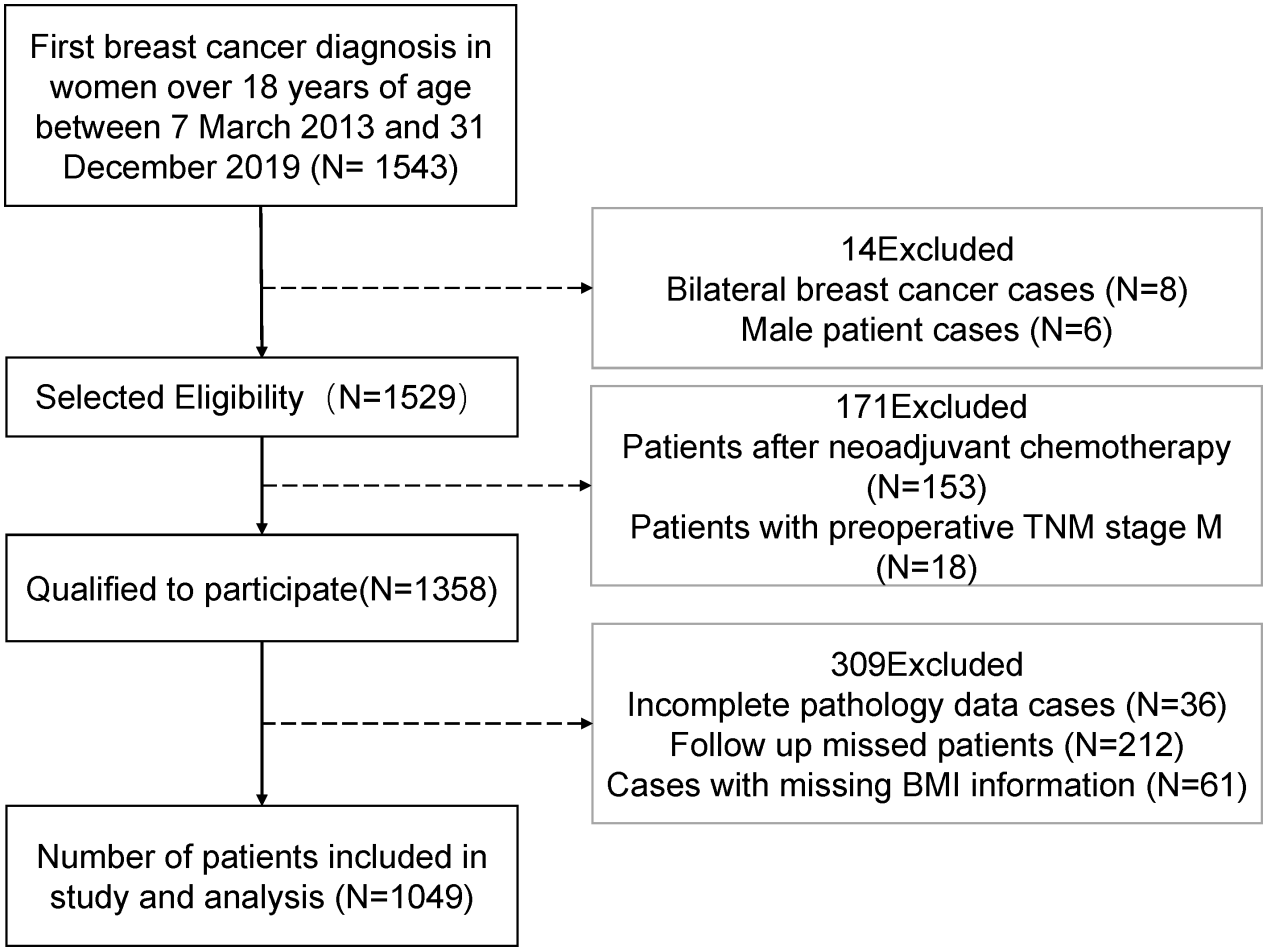
Flowchart for the data screening.

The retrospective cohort research entailed hospitalized Adult female patients(age>18)who identified with a diagnosis of BC at the People’s Hospital of Guangxi Zhuang Autonomous Region, China, from March 7, 2013 through December 31, 2019 (registration site http://www.chictr.org.cn/index.aspx; registration number ChiCTR2200058542). For this investigation, a large sample of 1543 patients’ records were chosen, and only 1049 breast cancer patients retained the complete data required to be included in the study. Seven criteria were used to exclude patients: cases with missing BMI data, male BC patients, patients with bilateral BC, BC patients with neoadjuvant chemotherapy, patients with missing pathological data, patients were not successfully followed up, patients with TNM stage M or patients with preoperative stage IV. Patients were included and excluded according to the criteria as shown in **Figure 1**.

Our research included the following patients, followed up from 2013 to 2019. Primarily, their survival status, survival time, and relevant information needed for this study were collected through telephone contact and patient outpatient review. Patients were seen at least every three months for the first three years, and then every six months after that. At each follow-up visit, the physician performed a physical examination or obtained a detailed medical history. At each physical examination or clinical suspicion of neoplasms recurrence or metastasis, relevant imaging examinations were performed regularly, and cytology was performed if necessary.

BMI at diagnosis was defined according to the World Health Organization (WHO) standard: the underweight, <18.5 kg/m^2^; normal, 18.5 - 24.9 kg/m^2^; overweight, 25.0 – 29.9 kg/m^2^; and obese, ≥30 kg/m^2^ (15). Overall survival was indicated from the time of diagnosis until the time of death. The BCSS is defined as the period of time (in months) between breast cancer diagnosis and cancer death or end of follow-up. This study protocol complied with the ethical guidelines of the Declaration of Helsinki (6th revision, 2008) and was approved by the Ethics Committee of the People’s Hospital of Guangxi Zhuang Autonomous Region, China. Individual informed consent was not obtained for this study because we used anonymized electronic medical record data as a summary analysis and no personal health data were available.

### 2.2 Statistical method

For continuous variables, medians and interquartile ranges (IQRs) as well as frequencies (percentages) were used for descriptive analysis to describe the data on baseline characteristics. Baseline characteristics were compared between groups with the Chi-square test for categorical data and the Wilcoxon rank sum test for continuous data. Multivariate Cox regression for assessing the influence of BMI on OS and BCSS with adjustment for the impact of independent variables (age, histopathological grade, tumor size, LN positivity, KI67 level, ER status, adjuvant therapy, PR status, Her2 status, surgical modality, hormonal therapy) was employed. The raw and adjusted hazard ratios (HRs) and 95% confidence intervals (CIs) for OS and BCSS were calculated based on BMI levels. Potential nonlinear associations were examined by modelling BMI levels using restricted cubic spline curves (RCS), adjusting for potential confounding factors, and checking if the independent variables are classed as intervals using a smooth curve fit.

Segmented regression (also referred to as “segment-by-segment regression”) is then performed by fitting each interval using a separate line segment. P-values for the nonlinearity of the smooth profile fit were computed by making a log-likelihood ratio examination of the single-linear (non-segmented) model in comparison with the segmented regression model. Determine the threshold level of BMI once the turning point delivers the maximum model likelihood.

All of the analyzed were carried out by means of the adherence to the below statistical package: R 3.4.3 (http://www.R-project.org, The R Foundation). Statistical significance was established at P<0.05 with a two-sided exam.

## 3 Results

### 3.1 Demographic and clinical characteristics of patients

A grand total of 1049 female BC patients met the analysis criteria. All diagnosed patients were women older than 18 years, with a median age at diagnosis of 51.00 (interquartile range: 44.00–59.00) years. In **Table 1**, patients who are classified by their body mass index are shown their baseline clinicopathologic characteristics. There were statistically significant variations in age, molecular subtype, OS, and BCSS across the BMI-classified groups. Underweight or normal-weight patients were significantly more likely to be young and develop luminal B breast cancer (all P<0.05).

**Table 1:** Baseline Characteristics of Patients Undergoing Breast Cancer Surgery Categorized by Body Mass Index (BMI) Category.

Characteristic	Total	underweight	normal weight	overweight	obese	
		<18.5 kg/m^2^	18.5-24.9 kg/m^2^	25-29.9 kg/m^2^	≥30 kg/m^2^	P-value
**No. of subjects**	1049	42	589	335	83	
Age (years)						
**Median (IQR)**	51.00 (44.00-59.00)	48.50 (39.25-58.00)	49.00 (43.00-57.00)	53.00 (47.00-62.00)	58.00 (52.00-65.00)	<0.001
<45	286 (27.26%)	16 (38.10%)	198 (33.62%)	64 (19.10%)	8 (9.64%)	<0.001
**>=45**	763 (72.74%)	26 (61.90%)	391 (66.38%)	271 (80.90%)	75 (90.36%)	
**Follow-up time (years)**	4.87 (3.26-6.84)	5.40 (3.10-6.58)	4.99(3.33-7.02)	4.57 (3.22-6.63)	4.45 (3.16-6.20)	0.090
**Tumor size(cm)**						0.112
<2cm	462 (44.04%)	16 (38.10%)	271 (46.01%)	136 (40.60%)	39 (46.99%)	
>2cm	534 (50.91%)	21 (50.00%)	285 (48.39%)	187 (55.82%)	41 (49.40%)	
Unknown	53 (5.05%)	5 (11.90%)	33 (5.60%)	12 (3.58%)	3 (3.61%)	
**Histopathological grade**						0.304
Low, intermediate (G1, G2)	612 (58.34%)	24 (57.14%)	351 (59.59%)	184 (54.93%)	53 (63.86%)	
High, G3	258 (24.59%)	8 (19.05%)	135 (22.92%)	94 (28.06%)	21 (25.30%)	
Unknown	179 (17.06%)	10 (23.81%)	103 (17.49%)	57 (17.01%)	9 (10.84%)	
**LN positive N (%)**						0.312
Negative	588 (56.05%)	28 (66.67%)	341 (57.89%)	175 (52.24%)	44 (53.01%)	
Positive	437 (41.66%)	14 (33.33%)	232 (39.39%)	154 (45.97%)	37 (44.58%)	
Unknown	24 (2.29%)	0 (0.00%)	16 (2.72%)	6 (1.79%)	2 (2.41%)	
**Molecular subtype**						0.009
Luminal A	255 (24.31%)	8 (19.05%)	142 (24.11%)	88 (26.27%)	17 (20.48%)	
Luminal B	371 (35.37%)	19 (45.24%)	185 (31.41%)	125 (37.31%)	42 (50.60%)	
Her2+	121 (11.53%)	3 (7.14%)	71 (12.05%)	36 (10.75%)	11 (13.25%)	
Her2-	133 (12.68%)	3 (7.14%)	89 (15.11%)	36 (10.75%)	5 (6.02%)	
Triple negative	120 (11.44%)	6 (14.29%)	69 (11.71%)	42 (12.54%)	3 (3.61%)	
Unknown	49 (4.67%)	3 (7.14%)	33 (5.60%)	8 (2.39%)	5 (6.02%)	
**KI67 level**						0.126
<14%	293 (27.93%)	7 (16.67%)	167 (28.35%)	99 (29.55%)	20 (24.10%)	
14%≤1 ≤ 30%	401 (38.23%)	22 (52.38%)	222 (37.69%)	120 (35.82%)	37 (44.58%)	
>30%	308 (29.36%)	9 (21.43%)	169 (28.69%)	107 (31.94%)	23 (27.71%)	
Unknown	47 (4.48%)	4 (9.52%)	31 (5.26%)	9 (2.69%)	3 (3.61%)	
**ER status**						0.088
Negative	289 (27.55%)	10 (23.81%)	178 (30.22%)	88 (26.27%)	13 (15.66%)	
Positive	748 (71.31%)	32 (76.19%)	402 (68.25%)	245 (73.13%)	69 (83.13%)	
Unknown	12 (1.14%)	0 (0.00%)	9 (1.53%)	2 (0.60%)	1 (1.20%)	
**PR status**						0.193
Negative	396 (37.75%)	15 (35.71%)	232 (39.39%)	128 (38.21%)	21 (25.30%)	
Positive	638 (60.82%)	27 (64.29%)	346 (58.74%)	204 (60.90%)	61 (73.49%)	
Unknown	15 (1.43%)	0 (0.00%)	11 (1.87%)	3 (0.90%)	1 (1.20%)	
**Her2 status**						0.118
Negative	754 (71.88%)	34 (80.95%)	406 (68.93%)	252 (75.22%)	62 (74.70%)	
Positive	251 (23.93%)	6 (14.29%)	159 (26.99%)	71 (21.19%)	15 (18.07%)	
Unknown	44 (4.19%)	2 (4.76%)	24 (4.07%)	12 (3.58%)	6 (7.23%)	
**Operation method**						0.097
Conserving surgery	168 (16.02%)	4 (9.52%)	107 (18.17%)	49 (14.63%)	8 (9.64%)	
Mastectomy	881 (83.98%)	38 (90.48%)	482 (81.83%)	286 (85.37%)	75 (90.36%)	
**Adjuvant therapy**						0.067
Chemotherapy	474 (45.19%)	18 (42.86%)	273 (46.35%)	149 (44.48%)	34 (40.96%)	
Radiotherapy	34 (3.24%)	0 (0.00%)	24 (4.07%)	5 (1.49%)	5 (6.02%)	
Both	262 (24.98%)	6 (14.29%)	148 (25.13%)	86 (25.67%)	22 (26.51%)	
None	279 (26.60%)	18 (42.86%)	144 (24.45%)	95 (28.36%)	22 (26.51%)	
**Hormonal therapy N (%)**						0.140
No	416 (39.66%)	17 (40.48%)	242 (41.09%)	134 (40.00%)	23 (27.71%)	
Yes	633 (60.34%)	25 (59.52%)	347 (58.91%)	201 (60.00%)	60 (72.29%)	
**OS**						0.023
No	978 (93.23%)	38 (90.48%)	556 (94.40%)	313 (93.43%)	71 (85.54%)	
Yes	71 (6.77%)	4 (9.52%)	33 (5.60%)	22 (6.57%)	12 (14.46%)	
**BCSS**						0.001
No	978 (95.14%)	38 (92.68%)	556 (96.53%)	313 (95.14%)	71 (86.59%)	
Yes	50 (4.86%)	3 (7.32%)	20 (3.47%)	16 (4.86%)	11 (13.41%)	

### 3.2 Non-linear relationship of BMI with OS and BCSS

**Figure 2:**
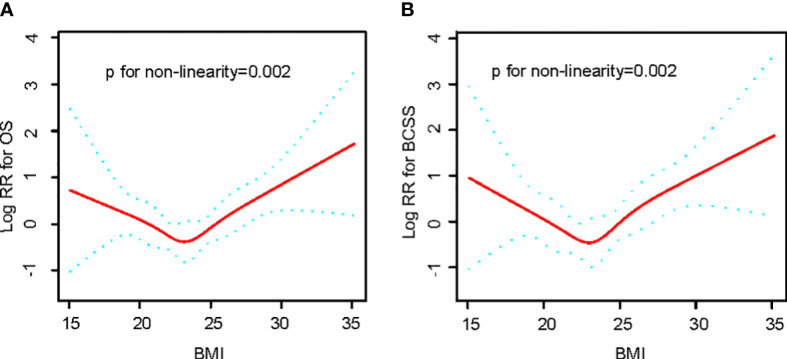
Relationships between BMI and the probability of OS **(A)** and BCSS **(B)** Non-linear associations between BMI and OS and BCSS were found (P < 0.05). Solid line and dashed lines represent estimated values and their corresponding 95% CIs. Adjusted for age, tumor size, histopathological grade, LN positive, KI67 level, ER status, PR status, Her2 status, operation method, adjuvant therapy, hormonal therapy.

Observations show a U-curve Connection between BMI level and OS or BCSS by the adjusted smoothing of the curve fit (**Figure 2**). Meanwhile, threshold effect analysis (**Table 2**) demonstrated a significant U-curve association between BMI and OS and BSCC (P<0.05 for log-likelihood ratio test). The turning point for BMI was identified as 23 kg/m^2^ on OS and BCSS. On the left side of the turning point, the risk of OS (fully adjusted HR, 0.83; 95% CI, 0.70, 0.98) and BCSS (fully adjusted HR, 0.80; 95% CI, 0.65, 0.98) were adversely correlated with BMI. In contrast, to the right of the turning point, the risk of OS (fully adjusted HR, 1.22; 95% CI, 1.10, 1.37) and BCSS (fully adjusted HR, 1.28; 95% CI, 1.13, 1.46) was positively related to BMI.

**Table 2:** Threshold effect analysis of BMI on OS and BCSS among 1049 breast patients.

Variable	Model 1		Model 2		Model 3	
	HR (95% CI)*	P-value	HR (95% CI)*	P-value	HR (95% CI)*	P-value
OS						
Inflection point★						
BMI<23 kg/m^2^	0.87 (0.74, 1.02)	0.085	0.85 (0.72, 1.00)	0.046	0.83 (0.70, 0.98)	0.029
BMI>23 kg/m^2^	1.18 (1.07, 1.30)	0.001	1.18 (1.07, 1.31)	0.002	1.22 (1.10, 1.37)	<0.001
P for log likelihood ratio test		0.012		0.007		0.002
BCSS						
Inflection point★						
BMI<23 kg/m^2^	0.85 (0.70, 1.03)	0.106	0.82 (0.68, 1.00)	0.054	0.80 (0.65, 0.98)	0.030
BMI>23 kg/m^2^	1.21 (1.09, 1.36)	<0.001	1.23 (1.09, 1.39)	<0.001	1.28 (1.13, 1.46)	<0.001
P for log likelihood ratio test		0.014		0.007		0.002

Model 1: unadjusted. Model 2: adjusted for tumor size, age, histopathological grade. Model 3: adjusted for tumor size, age, histopathological grade, LN positive, KI67 level, ER status, PR status, Her2 status, operation method, adjuvant therapy, hormonal therapy.

★Fitting model by two-piecewise Cox proportional hazards model.

### 3.3 Association of BMI with OS and BCSS

**Figure 3:**
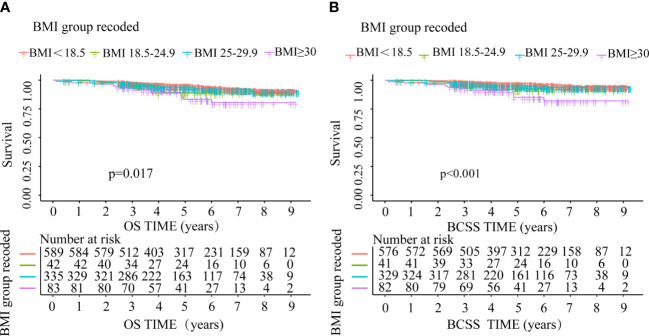
OS and BCSS comparison between underweight, normal weight, overweight and obese group **(A)** OS, **(B)** BCSS.

During the follow-up period after BC diagnosis (median 4.87 years, interquartile range 3.26-6.84 years), 71 BC patients (6.77%) died, of which 50 (70.42%) were attributed to BC. The Kaplan-Meier outcomes indicated that BC cases with normal weight had a superior survival rate than the rest of an alternative group (**Figure 3**). Compared with patients with normal weight, the risks for OS and BCSS were higher among those in underweight, overweight and obese group. However, OS and BCSS were non-significantly higher in underweight and overweight group (**Table 3**).

**Table 3:** Cox’s proportional hazard regression model for overall survival (OS) and breast cancer specific survival (BCSS).

Characteristic	Overall survival						Breast cancer specific survival					
	Alive	Death	HR (95% CI)	p value	aHR (95% CI)	p value	Alive	Death	HR (95% CI)	p value	aHR (95% CI)	p value
Age(years)												
<45	269 (27.51%)	17 (23.94%)	1		1		269 (27.51%)	13 (26.00%)	1		1	
>=45	709 (72.49%)	54 (76.06%)	1.23 (0.71, 2.12)	0.461	1.14 (0.64, 2.04)	0.656	709 (72.49%)	37 (74.00%)	1.10 (0.59, 2.07)	0.763	0.90 (0.45, 1.78)	0.756
BMI classification												
underweight	38 (3.89%)	4 (5.63%)	1.76 (0.62, 4.96)	0.286	1.92 (0.65, 5.65)	0.236	38 (3.89%)	3 (6.00%)	2.19 (0.65, 7.37)	0.206	2.38 (0.68, 8.38)	0.177
normal weight	556 (56.85%)	33 (46.48%)	1		1		556 (56.85%)	20 (40.00%)	1		1	
overweight	313 (32.00%)	22 (30.99%)	1.25 (0.73, 2.14)	0.424	1.19 (0.68, 2.09)	0.534	313 (32.00%)	16 (32.00%)	1.48 (0.76, 2.85)	0.246	1.38 (0.70, 2.73)	0.352
obese	71 (7.26%)	12 (16.90%)	2.78 (1.43, 5.38)	0.003	3.21 (1.55, 6.62)	0.002	71 (7.26%)	11 (22.00%)	4.11 (1.97, 8.58)	<0.001	4.87 (2.15, 11.05)	<0.001
Tumor size(cm)												
<2cm	444 (45.40%)	18 (25.35%)	1		1		444 (45.40%)	11 (22.00%)	1		1	
>2cm	482 (49.28%)	52 (73.24%)	2.43 (1.42, 4.16)	0.001	1.70 (0.97, 2.97)	0.062	482 (49.28%)	39 (78.00%)	3.01 (1.54, 5.89)	0.001	1.88 (0.94, 3.79)	0.075
Unknown	52 (5.32%)	1 (1.41%)	0.45 (0.06, 3.36)	0.436	0.28 (0.04, 2.20)	0.228	52 (5.32%)	0 (0.00%)	Inf	0.996	Inf	0.997
Histopathological grade												
Low, intermediate (G1, G2)	579 (59.20%)	33 (46.48%)	1		1		579 (59.20%)	21 (42.00%)	1		1	
High, G3	236 (24.13%)	22 (30.99%)	1.66 (0.97, 2.85)	0.065	1.24 (0.67, 2.27)	0.491	236 (24.13%)	18 (36.00%)	2.11 (1.12, 3.96)	0.020	1.52 (0.75, 3.07)	0.245
Unknown	163 (16.67%)	16 (22.54%)	1.61 (0.88, 2.92)	0.120	2.06 (1.07, 3.98)	0.031	163 (16.67%)	11 (22.00%)	1.75 (0.84, 3.63)	0.133	2.41 (1.06, 5.49)	0.036
LN positive N (%)												
Negative	561 (57.36%)	27 (38.03%)	1		1		561 (57.36%)	17 (34.00%)	1		1	
Positive	393 (40.18%)	44 (61.97%)	2.35 (1.45, 3.79)	0.001	2.77 (1.65, 4.65)	<0.001	393 (40.18%)	33 (66.00%)	2.79 (1.55, 5.00)	0.001	3.29 (1.73, 6.25)	<0.001
Unknown	24 (2.45%)	0 (0.00%)	Inf	0.995	Inf		24 (2.45%)	0 (0.00%)	Inf	0.996	Inf	0.996
Molecular subtype												
Luminal A	243 (24.85%)	12 (16.90%)	1				243 (24.85%)	7 (14.00%)				
Luminal B	349 (35.69%)	22 (30.99%)	1.18 (0.58, 2.39)	0.643			349 (35.69%)	19 (38.00%)				
Her2+	112 (11.45%)	9 (12.68%)	1.54 (0.65, 3.67)	0.325			112 (11.45%)	6 (12.00%)				
Her2-	122 (12.47%)	11 (15.49%)	1.69 (0.75, 3.83)	0.209			122 (12.47%)	8 (16.00%)				
Triple negative	109 (11.15%)	11 (15.49%)	1.80 (0.80, 4.09)	0.158			109 (11.15%)	6 (12.00%)				
Unknown	43 (4.40%)	6 (8.45%)	2.00 (0.75, 5.34)	0.169			43 (4.40%)	4 (8.00%)				
KI67 level												
<14%	279 (28.53%)	14 (19.72%)	1		1		279 (28.53%)	9 (18.00%)	1		1	
14%≤1 ≤ 30%	380 (38.85%)	21 (29.58%)	1.08 (0.55, 2.13)	0.820	0.86 (0.42, 1.76)	0.686	380 (38.85%)	18 (36.00%)	1.44 (0.65, 3.20)	0.375	1.06 (0.45, 2.47)	0.893
>30%	279 (28.53%)	29 (40.85%)	1.89 (1.00, 3.59)	0.050	1.29 (0.63, 2.64)	0.492	279 (28.53%)	20 (40.00%)	2.06 (0.94, 4.52)	0.072	1.16 (0.48, 2.79)	0.737
Unknown	40 (4.09%)	7 (9.86%)	2.53 (1.02, 6.29)	0.046	1.90 (0.63, 5.68)	0.253	40 (4.09%)	3 (6.00%)	1.81 (0.49, 6.70)	0.374	0.74 (0.10, 5.42)	0.768
ER status												
Negative	265 (27.10%)	24 (33.80%)	1		1		265 (27.10%)	16 (32.00%)	1		1	
Positive	703 (71.88%)	45 (63.38%)	0.73 (0.44, 1.20)	0.213	1.11 (0.50, 2.45)	0.802	703 (71.88%)	32 (64.00%)	0.77 (0.42, 1.40)	0.391	1.16 (0.47, 2.89)	0.746
Unknown	10 (1.02%)	2 (2.82%)	2.11 (0.50, 8.92)	0.311	Inf		10 (1.02%)	2 (4.00%)	3.10 (0.71, 13.51)	0.131	Inf	0.999
PR status												
Negative	362 (37.01%)	34 (47.89%)	1		1		362 (37.01%)	26 (52.00%)	1		1	
Positive	603 (61.66%)	35 (49.30%)	0.63 (0.39, 1.01)	0.056	0.73 (0.37, 1.41)	0.347	603 (61.66%)	22 (44.00%)	0.52 (0.29, 0.91)	0.023	0.51 (0.24, 1.07)	0.076
Unknown	13 (1.33%)	2 (2.82%)	1.63 (0.39, 6.79)	0.502	Inf		13 (1.33%)	2 (4.00%)	2.09 (0.50, 8.82)	0.315	Inf	
Her2 status												
Negative	707 (72.29%)	47 (66.20%)	1		1		707 (72.29%)	34 (68.00%)	1		1	
Positive	232 (23.72%)	19 (26.76%)	1.23 (0.72, 2.09)	0.454	0.98 (0.55, 1.75)	0.941	232 (23.72%)	13 (26.00%)	1.17 (0.62, 2.21)	0.637	0.82 (0.41, 1.64)	0.571
Unknown	39 (3.99%)	5 (7.04%)	1.56 (0.62, 3.93)	0.344	0.91 (0.27, 3.09)	0.884	39 (3.99%)	3 (6.00%)	1.34 (0.41, 4.36)	0.631	0.51 (0.07, 3.90)	0.518
Operation method												
Conserving surgery	160 (16.36%)	8 (11.27%)	1		1		160 (16.36%)	4 (8.00%)	1		1	
Mastectomy	818 (83.64%)	63 (88.73%)	1.39 (0.66, 2.89)	0.384	0.85 (0.40, 1.83)	0.680	818 (83.64%)	46 (92.00%)	2.04 (0.74, 5.68)	0.170	1.30 (0.45, 3.75)	0.628
Adjuvant therapy												
Chemotherapy	431 (44.07%)	43 (60.56%)	1		1		431 (44.07%)	30 (60.00%)	1		1	
Radiotherapy	31 (3.17%)	3 (4.23%)	1.03 (0.32, 3.31)	0.965	0.93 (0.27, 3.14)	0.903	31 (3.17%)	2 (4.00%)	0.98 (0.23, 4.10)	0.978	0.69 (0.15, 3.13)	0.634
Both	255 (26.07%)	7 (9.86%)	0.33 (0.15, 0.74)	0.007	0.32 (0.14, 0.71)	0.006	255 (26.07%)	5 (10.00%)	0.33 (0.13, 0.86)	0.023	0.29 (0.11, 0.76)	0.012
None	261 (26.69%)	18 (25.35%)	0.75 (0.43, 1.30)	0.302	0.70 (0.37, 1.34)	0.282	261 (26.69%)	13 (26.00%)	0.77 (0.40, 1.48)	0.437	0.75 (0.35, 1.61)	0.455
Hormonal therapy N (%)												
No	382 (39.06%)	34 (47.89%)	1		1		382 (39.06%)	23 (46.00%)	1		1	
Yes	596 (60.94%)	37 (52.11%)	0.73 (0.46, 1.16)	0.181	0.80 (0.41, 1.54)	0.504	596 (60.94%)	27 (54.00%)	0.77 (0.44, 1.34)	0.354	0.94 (0.43, 2.08)	0.885

OS overall survival, BCSS breast cancer specific survival, HR hazard ratio, aHR adjusted hazard ratio, CI confidence interval.

Data presented as n (%) and HR (95% CI).

HRs are unadjusted or adjusted based on Cox’s proportional-hazard regression models.

Patient age; tumor size; histopathological grade, LN positive, KI67 level, ER status, PR status, Her2 status, operation method, adjuvant therapy, hormonal therapy included in the multivariate analysis model.

## 4 Discussion

In our group analysis, Breast cancer risk is independently correlated with BMI, and we found that the OS and BCSS of underweight and obese BC patients were meaningfully lower than those of breast cancer patients with normal BMI, suggesting a U-shaped correlation between the two, and we found that the inflection point of BMI of the U-shaped curve was 23kg/m^2^. Similar to recent relevant studies (15–17), we found significant differences among groups classified by age, BMI, molecular subtypes, OS and BCSS. We regard that this is a valuable study showing that breast cancer patients with reasonable control of the breast cancer patients who fall within the normal BMI range show a better prognosis.

Our results further confirm the previous study, where a study of 8,394 women with breast cancer in western China found BMI and DFS have a U-shaped association, whereas the difference in DFS between obese and normal weight premenopausal patients was not statistically significant (18). Furthermore, Ye won-jeon et al. analyzed data from 4,021 South Korean patients with invasive BC and discover a U-curve connection between BMI and mortality across the Enrolled patients, with underweight and obese people having inferior OS and BCSS compared to normal weight people (19). In addition, a report of 4062 BC patients from the Shanghai research institute showed a U-shaped relationship between overall and central obesity and late all-cause mortality in long-term BC survivors when using post-diagnostic BMI and waist-to-hip ratio (WHR) as indexes (20). However, significant confounding variables such as Ki67 and P53 status, nuclear grade, socioeconomic status, and anti-HER2 therapy may be absent from these studies, which may influence the results (17, 21, 22). In the current study, the complete required data population was included, and the influence of independent variables (age, tumor size, histopathological grade, LN positive, KI67 level, ER status, PR status, Her2 status, surgical method, adjuvant therapy, hormone therapy) was adjusted. Thus, a more reasonable curve relationship can be obtained that more accurately expresses the correlation between BMI and BC prognosis.

Our study proposed the problem of curve inflection point for the first time, and analyzed the different trends around the fitting curve inflection point. The relationship between BMI and OS and BCSS is more accurately depicted with more accurate curves, which assists in identifying the relationship between BMI and prognosis. Won Kyung Cho et al. showed in a study of 5668 patients undergoing radical breast cancer surgery that BMI≥25 was a poor prognostic factor, and patients with BMI<25 had higher DFS and OS than those with BMI≥25 (P = 0.012 and 0.005, respectively) (23). At the right inflection point of the U-shaped curve (BMI > 23kg/m^2^), BMI was negatively correlated with BC prognosis. This curvilinear trend can be explained by the fact that obesity is associated with increased androgen precursor to estradiol peripheral conversion due to increased aromatase activities in bulky adipose tissue and reduced sex hormone binding globulin (24). Obesity also increases insulin and insulin-like growth factor and obesity-related regulatory proteins (25). In addition, obesity facilitates the accumulated PD-1+CD8+ depleted T cells in tumors and PD-1+CD8+ T cells are the main source of osteoclastin (OPN), which can mediate tumor progression by regulating multiple pathways (26). Therefore, highly circulating biological estrogens, growth cofactors and regulatory proteins may have a carcinogenic impact and promote neoplasms phenotype and development, thus adversely affecting the prognosis (19). Then it also accords with the majority of research’ conclusions that increased body mass index and obesity can have an effect on the prognosis of breast cancer (17, 27–29).

At the left of the inflection point of the U-shaped curve (BMI < 23kg/m^2^), BMI is positively correlated with the prognosis of BC, and the prognosis of BC corresponding to BMI near the inflection point is the best. The association between low BIM and decrease in survival can be explained, at least in part, by the existence of circulating tumor cells (CTCs) in the peripheral blood of BC patients. Low BMI may be associated with lower body composition factors and a weaker immune system, which may create a more favorable environment for CTCs to survive and establish secondary tumors, while low BMI and malnutrition are also associated with tumor activation pathways that promote the survival, invasion, and metastasis of CTCs (19, 30, 31). In addition, alterations in CTCs may affect oncology development and the effectiveness of the whole system antineoplastic therapy (32). Actually, low BMI could be related to malnutrition that could be related to advanced metastatic stage of BC with related alterations of immune system and associated inflammation (33). These mechanisms could explain the relationship between low BMI and poor prognosis in a subset of BC. Related clinical studies recommend intervention with BMI in the normal range to improve breast cancer prognosis (34–37).

We used data from 1049 participants as being part of a large sample study. It is however important to acknowledge several limitations of the study. In the first place, most of our research is based on looking backward, which could have led to sample bias. More prospective researches are still necessary to show the consensus of our findings. Second, patients were not surveyed for waist circumference, which is a potentially moderating factor in the interaction between BMI and BC characteristics (38, 39). Third, we were unable to collect data on the menstrual status of BC patients, which is associated with the long-term prognosis of BC patients. A woman’s menstrual status can be adjusted as she ages. In addition, we cannot differentiate whether a particularly sick person’s a low BMI is a consequence of malnutrition because of disease or a health disposition in the absence of malnutrition. However, even clinically, this can be difficult to distinguish. Lastly, it belongs to a monocentric study. It is possible that these factors may have an undetected impact on our results.

In conclusion, in our retrospective study, we observed that BMI was an independently prognostic factor in BC patients, with a U-shaped association with OS and BCSS levels. The connection between BMI and prognosis deserves more prospective studies in which clinical intervention of patients’ BMI can be performed according to the inflection point of U-shaped curve to improve the prognosis.

## Data availability statement

The raw data supporting the conclusions of this article will be made available by the authors, without undue reservation.

## Ethics statement

The project was conducted in accordance with the Declaration of Helsinki. The ethics committee of the Guangxi Zhuang Autonomous Region People’s Hospital approved the study (Ethics-KY-QT208 202205). Written informed consent was waived by the ethics committee of the Guangxi Zhuang Autonomous Region People’s Hospital due to the retrospective observational nature of the study.

## Author contributions

WSW and QQZ conceptualized and engineered the study and have reviewed the manuscript. All authors were involved in the data analysis and produced the first version of the manuscript. All authors contributed to the article and approved the submitted version.
